# Carry-Over of Aflatoxin B1 to Aflatoxin M1 in High Yielding Israeli Cows in Mid- and Late-Lactation

**DOI:** 10.3390/toxins5010173

**Published:** 2013-01-16

**Authors:** Malka Britzi, Shmulik Friedman, Joshua Miron, Ran Solomon, Olga Cuneah, Jakob A. Shimshoni, Stefan Soback, Rina Ashkenazi, Sima Armer, Alan Shlosberg

**Affiliations:** 1 National Residue Control Laboratory, Kimron Veterinary Institute, Bet Dagan 50250, Israel; E-Mails: malkab@moag.gov.il (M.B.); stefans@moag.gov.il (S.S.); 2 Israeli Dairy Board, Yehud 56470, Israel; E-Mail: shmulik@milk.org.il; 3 Department of Ruminant Science, Agricultural Research Organization, Bet Dagan 50250, Israel; E-Mail: jmiron@volcani.agri.gov.il; 4 Ambar Feed Production Company, Gan Shmuel 38810, Israel; E-Mail: ransal@shaham.moag.gov.il; 5 Department of Toxicology, Kimron Veterinary Institute, Bet Dagan 50250, Israel; E-Mails: olgacu@moag.gov.il (O.C.); jakobshimshoni@gmail.com (J.A.S.); 6 Department of Chemistry, Plant Protection and Inspection Services, Bet Dagan 50250, Israel; E-Mails: rinaa@moag.gov.il (R.A.); simaa@moag.gov.il (S.A.)

**Keywords:** aflatoxin B1, aflatoxin M1, carry-over, Israeli-Holstein cows

## Abstract

The potent hepatotoxin and carcinogen aflatoxin B1 (AFB1) is a common mycotoxin contaminant of grains used in animal feeds. Aflatoxin M1 (AFM1) is the major metabolite of AFB1 in mammals, being partially excreted into milk, and is a possible human carcinogen. The maximum permitted concentration of AFM1 in cows’ milk is 0.05 μg/kg in Israel and the European Union. Since milk yield and the carry-over of AFB1 in the feed to AFM1 in the milk are highly correlated, it was considered important to determine the AFM1 carry-over in Israeli-Holstein dairy cows, distinguished by world record high milk production. Twelve such cows were used to determine AFM1 carry-over following daily oral administration of feed containing ~86 μg AFB1 for 7 days. The mean carry-over rate at steady-state (Days 3–7) was 5.8% and 2.5% in mid-lactation and late-lactation groups, respectively. The carry-over appears to increase exponentially with milk yield and could be described by the equation: carry-over% = 0.5154 e^0.0521 × milk yield^, with *r*^2^ = 0.6224. If these data truly reflect the carry-over in the national Israeli dairy herd, the maximum level of AFB1 in feed should not exceed 1.4 μg/kg, a value 3.6 times lower than the maximum residue level currently applied in Israel.

## 1. Introduction

Aflatoxins (AF) are hepatotoxic and carcinogenic secondary metabolic products from fungi belonging in particular to the *Aspergillus flavus* and *A. parasiticus* species [1,2,3,4].

More than 20 AF-like secondary metabolites have been identified and aflatoxin B1 (AFB1) was shown to possess the most toxic and carcinogenic properties to humans and animals [2,3]. *A. flavus* and *A. parasiticus* strains that produce AF may develop under certain climatic conditions mainly in tropical and subtropical climates [1,2,3,4]. AF are found as natural contaminants in many feedstuffs of plant origin, especially in cereals but also in fruits, hazelnuts, almonds and foods consisting of, or manufactured from, these products and intended for human or animal consumption [1]. It is well known that AFB1 can cause chronic diseases in humans and animals and can have different effects such as hepatotoxicity, genotoxicity and immunotoxicity [2,3]. It has been estimated that more than 5 billion people in developing countries worldwide are at risk of chronic exposure to AFB1 through contaminated foods [5,6,7]. The primary disease associated with AFB1 intake is hepatocellular carcinoma, being the third-leading cause of death from cancer globally [8], with about 550,000–600,000 new cases each year [5,9] and aflatoxin may play a causative role in up to 28% of all global cases of hepatocellular carcinoma [5].

Upon ingestion by ruminants, AFB1 is partially destroyed in the rumen, whereas the absorbed AFB1 rapidly undergoes metabolic processes in the liver to various secondary metabolites [4,10,11]. Aflatoxin M1 (AFM1), a possible human carcinogen [12], is the major oxidized metabolite of AFB1 and is excreted primarily in the urine and less so in the milk [13,14]. The EU applies a maximum residue level (MRL) of 0.05 μg AFM1/kg in ruminant milk, and some countries in Africa, Asia and Latin America also enforce this level [3,14,15]. In contrast, the USA, many South American and several Asian countries, adopt a MRL of 0.5 μg/kg AFM1 in milk [1,2,3,4]. Israeli regulations concerning the dairy industry are harmonized with the EU regulations and therefore a MRL of 0.05 μg/kg for AFM1 in milk is applied.

The ability of cattle to transform AFB1 in the feed to AFM1 in the milk has been examined in the past in many studies, which demonstrated that such carry-over in dairy cows milked 2 times daily was usually 1%–2% of the ingested AFB1 for low-yielding cows (<30 kg milk yield/day) and up to ~6% for high-yielding cows (>30 kg milk yield/day) [16,17,18,19,20,21,22]. The extent of carry-over was found to be directly correlated to the milk yield as well as to the days in lactation [17,19,20,22,23]. Cows in early lactation (2 to 4 weeks after calving) show highest milk yields and a higher carry-over rate than cows in late lactation (34 to 36 weeks after calving), when milk yield naturally declines [22]. Additional factors that have been shown to affect the carry-over rate (often in individual cows) were species difference, general health of the animal, hepatic biotransformation capacity, rate of ingestion and the integrity of the mammary alveolar cell membranes [20,22,23,24,25].

Israeli-Holstein cows, a high-yielding, disease-resistant breed, that constitute all dairy cows in Israel, had an average yield of 11,400 kg of milk/cow in 2011, compared with yields in the US (9331 kg), Japan (7497 kg), the European Union (6139 kg) and Australia (5601 kg), making Israel the highest milk producing country / cow in the world [26,27]. Previous carry-over studies have been conducted in cows milked twice daily, whereas the current study used cows milked thrice daily, as is done in larger herds in Israel and some other countries with intensive dairy industries. 

The estimation of the carry-over rate of AFM1 in national dairy herds is of major importance in order to determine the acceptable AFB1 intake in feed. The aim of the current study was to determine the AFB1 in feed to AFM1 in milk carry-over rate in Israeli-Holstein dairy cows, in order to assess evidence-based recommendations for standards regarding a MRL of AFB1 in the feed of dairy cows.

## 2. Results and Discussion

The cows in this study were examined before, during and at the end of the study, and no obvious adverse effects on health were observed. An AFB1 intake of 100 μg/kg body weight caused a reduction in milk yield in cows [16,28,29], but as the level of AFB1 intake in our study was very much lower (mean of 0.15 μg/kg body weight), no changes in milk yield were expected or seen. Milk somatic cell counts were normal (lower than 200,000/mL) in all cows at all milkings in the present trial. AFM1 carry-over in milk has been related to several factors such as udder health, expressed as high somatic cell counts [19,21,23], however, a recent study largely discounted high somatic cell count as a marker for increased carry-over [19]. High milk yield and an early stage of lactation have been identified as the main factors contributing to increased carry-over [19,22]. High milk yield is a phenotype of breeding for increased production, but is also dependent upon intensive husbandry and feeding, and is correlated directly with the stage of lactation, whereby newly-calved cows rapidly reach peak production, which naturally declines with increasing length of lactation.

As Israel exports some dairy products to the European Community and the Israeli regulations concerning the dairy industry are harmonized with the EU regulations, a MRL for AFM1 in milk of 0.05 μg/kg has been set [15]. The EU regulations are based on the principle of ALARA (as low as reasonably achievable). The USA regulations are based on a risk analysis, wherein a level 10 times higher than the EU level is not considered harmful to human health, which is in line with the World Health Organization [30]. However, levels of AFM1 exceeding the MRL have been detected in Israel in milk from individual farms on several occasions, although at concentrations that exceed the Israeli MRL only marginally. However, this poses a problem for some dairy farmers who may produce wholesome and healthy milk that occasionally is legally non-compliant in terms of AFM1 concentrations. Therefore, the determination of the carry-over rate of AFM1 in Israeli dairy herds is of major importance in order to produce relevant regulatory guidelines and standards concerning levels of AFB1 in animal feed.

**Figure 1:**
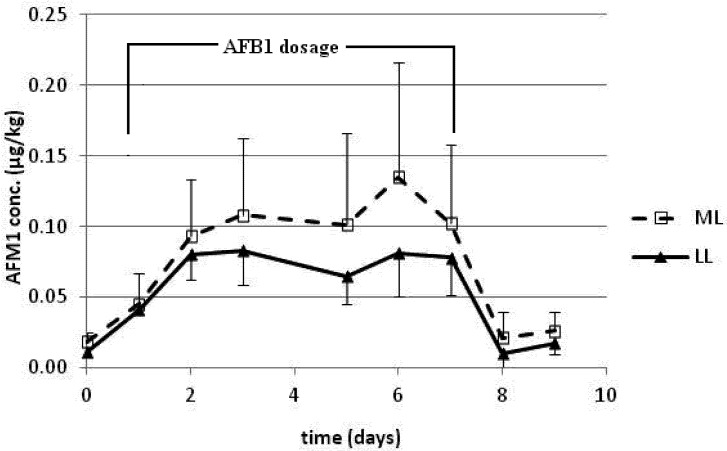
Mean aflatoxin M1 (AFM1) concentrations (μg/kg) in the milk of mid-lactation (ML) and late-lactation (LL) cows dosed with aflatoxin B1 (AFB1) for 7 days. Each day data point refers to the day of dosage and the concentration of AFM1 found in the milk in the three milkings thereafter. Data for Day 4 were not recorded.

Daily naturally occurring AFB1 residues in the total mixed ration (TMR) in this trial were a mean of 0.25 ± 0.05 μg/kg dry matter (DM). The non-spiked corn meal was found to contain < 1 μg/kg AFB1. With the 80 μg AFB1 spiked corn meal, the total mean daily intake of AFB1 was 86.2 ± 4.8 μg. The milk yields of each cow were recorded daily, with a mean of 44.7 ± 5.7 kg and 29.8 ± 2.6 kg for mid-lactation (ML) and late-lactation (LL) cows, respectively (Table 1). Daily concentrations of AFM1 in the bulk milk of the control cows fed TMR only were in the range of 0.010 (below the limit of quantification) to 0.013 μg/kg. In the cows given AFB1 the concentrations of AFM1 increased rapidly from the first day of AFB1 administration, in agreement with other studies [17,19], and reached a mean of 0.045 and 0.041 μg/kg in the milk of ML and LL cows, respectively, as depicted in Figure 1. The milk samples for day 4 were inadvertently mislabeled, and so we were unable to present reliable data for that day. A plateau in AFM1 concentrations was observed from the third day of administration with a mean of 0.113 and 0.075 μg/kg for ML and LL cows, respectively, and a steady state condition was maintained up to the last day of AFB1 administration. Previous studies have reported a plateau achieved by 24 h [18], 76 h [17], 7 days [19] and 9 days [24] following AFB1 ingestion. AFB1 intake and AFM1 excretion of each cow at steady-state are summarized in Table 1, indicating that the AFM1 concentrations during the period of AFB1 ingestion ranged between 0.06–0.21 μg/kg and 0.03–0.09 μg/kg for ML and LL cows, respectively. In a previous study [19], means of 0.059 and 0.062 μg/kg of AFM1 in milk were measured for high- and low-yielding cows, respectively, following administration of 98 μg AFB1. Depletion of AFM1 was also rapid in the present study, and one day after the last AFB1 administration, levels of AFM1 were lower than 0.05 μg/kg in all milk samples, similar to data from other studies [17,19]. 

**Table 1:** Aflatoxin intake and milk data of individual cows at steady-state conditions (Days 3–7).

Cow	AFB1 intake (μg/kg) ± SD	Milk yield (kg/day) ± SD	AFM1 concentration in milk (μg/kg) ± SD	AFM1 excretion in milk (μg/day) ± SD	Carry-over (%) ± SD
Mid-lactation					
1	86.4 ± 1.1	45.1 ± 1.5	0.13 ± 0.007	5.6 ± 0.1	6.4 ± 0.2
2	87.2 ± 1.3	41.7 ± 3.6	0.07 ± 0.012	3.2 ± 0.5	3.6 ± 0.5
3	87.4 ± 1.2	45.8 ± 1.4	0.12 ± 0.019	5.6 ± 1.0	6.4 ± 1.2
4	86.2 ± 1.5	48.4 ± 2.3	0.21 ± 0.046	10.3 ± 2.7	11.9 ± 3.3
5	86.5 ± 1.1	50.9 ± 2.2	0.06 ± 0.015	3.2 ± 0.7	3.6 ± 1.5
6	85.9 ± 1.8	35.1 ± 1.6	0.08 ± 0.036	2.6 ± 1.2	3.0 ± 0.9
Late-lactation					
7	87.4 ± 2.1	32.9 ± 2.1	0.09 ± 0.016	3.1 ± 0.7	3.5 ± 0.8
8	86.5 ± 2.4	29.8 ± 2.1	0.09 ± 0.018	2.6 ± 0.6	3.0 ± 0.8
9	87.5 ± 2.3	27.3 ± 1.5	0.06 ± 0.018	1.4 ± 0.4	1.6 ± 0.5
10	85.3 ± 2.3	26.3 ± 1.4	0.09 ± 0.016	2.3 ± 0.5	2.6 ± 0.6
11	86.7 ± 3.3	30.4 ± 2.7	0.07 ± 0.036	1.8 ± 0.8	2.1 ± 0.8
12	86.7 ± 1.6	30.8 ± 0.5	0.03 ± 0.016	1.4 ± 0.4	1.6 ± 0.5

**Figure 2:**
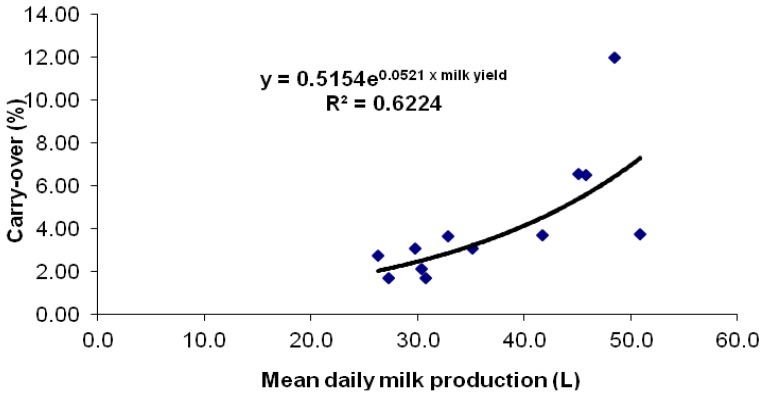
Mean milk yield per cow and mean AFB1 to AFM1 carry-over% of the 12 cows consuming ~86 μg aflatoxin B1 daily at steady-state (Days 3–7).

Figure 2 depicts the mean milk yields and the mean carry-over% pairs at steady-state (Days 3–7). The mean carry-over at steady state was 5.8% for ML cows (yield > 35 kg/day) and 2.5% for LL cows (yield < 33 kg/day) and was within the ranges reported by others [16,17,18,19,20,21,22]. When mean milk yield was paired with mean carry-over% in order to evaluate the correlation at steady state, the calculated linear equation was:

carry-over% = *y* = 0.2362 × milk yield − 4.5438, with *r*^2^ = 0. 508 (1)

However, an exponential regression yielded a better correlation, producing the following equation:

carry-over% = 0.5154 e^0. 0521 × milk yield^, with *r*^2^ = 0. 6224. (2)

As the population could not be assumed to be normally distributed, non-parametric statistics were applied. The observed correlation of mean carry-over% at steady-state with milk yield was extremely significant (*p* < 0.0001). It is worthy of note that the highest-yielding mid-lactation cow had a low carry-over, although higher than late-lactation cows (Table 1). It is interesting that one ML cow, # 4, had a carry-over of 11.9 ± 3.3, which is 204.6% above the mean for the ML group. In a previous trial [31], one of eight cows also had an exceptionally high carry-over of 155.3% above the mean. It is believed that these exceptions (“super AFM1 excretors”) may occur naturally in a particular dairy cow genetic pool and their higher carry-over rates may elevate the carry-over reported for that herd in which they contribute milk.

The mean AFM1 concentration in milk at steady state was 0.113 μg/kg for ML cows, being 2.26 times higher than the MRL of 0.05 μg/kg, while in LL cows the average AFM1 concentration was 0.075 μg/kg, being 1.5 times higher than the MRL. Consequently, based on the present study, the daily AFB1 exposure of the Israeli cow characterized by an average daily intake of 25 kg DM feed and an average daily milk yield of 45 kg during early lactation [26,27] has to be below 1.4 μg/kg in the feed in order to ensure milk production compliant with the regulatory MRL of 0.05 μg/kg AFM1 applied in Israel. The maximum AFB1 level in feed presently recommended in Israel for milking ruminants is 5 μg/kg. It remains to be assessed whether a regulatory change should be made with the feed MRL to match our findings, according to the ALARA principle. 

## 3. Materials and Methods

### 3.1. Animals and Treatment

The study was carried out utilizing 12 Israeli-Holstein cows housed at the Agricultural Research Organization Dairy Research Farm (Bet Dagan, Israel). The research and animal care protocols were approved (number IL346/11) by the Israeli Animal Experimentation Ethics Committee in accordance with the requirement of the Prevention of Cruelty to Animals (Animal Experimentation) Law, 1994.

From 2 weeks before the start of the experiment, all 12 milking cows in the study were fed the same total mixed ration (TMR) and blocked into 2 groups by daily milk production, comprising mid-lactation (ML) cows (8 to 20 weeks after calving) yielding 36 to 51 kg/day and late-lactation (LL) cows (33 to 46 weeks after calving) yielding 26 to 33 kg/day. The TMR was fed *ad libitum* once daily at 11.00 h and cows were milked 3 times daily at 6:00 a.m., 14:00 p.m. and 22:00 p.m. Cows were fed individually in their specific stalls and the weight of TMR ingested was monitored using a computerized system designed to electronically identify individual cows and their feed intake [32]. Daily dry matter (DM) intake of individual cows was determined based on the DM content of the TMR consumed daily. The diet was formulated according to the nutrient requirements of dairy cattle [33] for an average cow weighing 600 kg, 140 days in lactation and with a 35 kg milk yield (3.8% fat, 3.35% protein). The composition of the basic TMR is shown in Table 2. The 12 cows were individually given AFB1 daily for 7 days when, before the morning feed, a 500 g bolus of ground corn meal spiked with 80 μg AFB1 (Sigma, Rehovot, Israel) was offered to, and eaten by each cow. AFB1 levels in the corn were determined in two samples. TMR samples (200 g) were collected daily, dried at 60 °C in a ventilated oven until constant weight, ground to pass through a 1 mm sieve (Thomas-Wiley Laboratory Mill, PA, USA) and frozen until AFB1 analysis. AFB1 levels of the TMR were determined (see below) before and on each day of the experiment and the total daily AFB1 intake was calculated from the summation of the bolus dose and the daily TMR levels. 

**Table 2:** Constituents of the total mix ration (TMR) fed to the research herd during the study.

Ingredients	% of TMR (dry matter)
Wheat silage	22
Oat hay	5
Corn silage	7
Corn grain	13.7
Barley grain	6.58
Wheat grain	6.58
Sunflower meal	5.27
Canola meal	2.58
Corn gluten feed	14.4
DDGS ^1^	9.65
Whole cotton seeds	0.67
Molasses	2.48
NaHCO_3_	0.70
Ca-LCFA ^2^	1.53
NaCl + CaCl_2_	1.24
CaCO_3_	0.22
Urea	0.30
Minerals + vitamins mix ^3^	0.10

^1^ Dried distillers grains with soluble; ^2^ Calcium salts of long-chain fatty acids; ^3^ Containing (g/kg DM mix): Zn 24, Fe 24, Cu 12.8, Mn 24, I 1.44, Co 0.32, Se 0.32; 4.8 Vit. A; 0.08 Vit. D_3_; 0.03 Vit. E.

Milk yield (kg) was recorded daily for each cow by an automatic meter (Afimilk SAE, Kibbutz Afikim, Israel). Milk samples were collected from each cow at each milking for 10 consecutive days (during the first day of the pre-experimental period, 7 days of the experimental period and for 2 days after the AFB1 ingestion period). Samples from each day of milking (comprising aliquots from the combined three milkings after each dosage) were frozen for subsequent analysis of AFM1. Aliquots of milk samples were also stored at 4 °C in the presence of 2-bromo-2-nitropropane-1,3-diol, for somatic cell count using a Fossomatic 360 (Foss Electric, Hillerod, Denmark). Milk samples were taken from the bulk tank in the research herd from cows receiving the same TMR but without added AFB1, as a control group.

The daily milk yield of all cows given AFB1 was discarded into the sewage system and this continued for three days after the last dosage at which time the AFM1 levels were below the Israeli regulatory limit (0.005 μg/kg). Consequently, on the fourth day following the last AFB1 dosage, the cows were returned to their normal usage as dairy cows. 

### 3.2. AFB1 Assay in the Ground Corn Meal and the TMR

Ten grams of air-dried feeds were sonicated in 40 mL methanol in an ultrasonic bath for 20 min. The sample was then filtered through MN 615 filter paper. Twenty ml of phosphate buffered saline at pH 7.4 (P-5386, Sigma, Rehovot, Israel) were added to 5 mL of the filtrate and filtered through a glass filter (Munktell MGA, Bärenstein, Germany). 10 mL of the buffered filtrate were then loaded on a NeoColumn Aflatoxin narrow bore column (Neogen 8040, Lexington, NC, USA). The column was washed with 10 mL of methanol: water (25:75 *v*/*v*) and the sample was eluted with 1 mL of methanol followed by 1 mL of water. The eluent was filtered through a 0.22 μm nylon filter (Starlab Scientific SLSF13NY22, Xi’an, China) prior to HPLC analysis. The method utilized an Agilent 1200 (Agilent Technologies, Böblingen, Germany) liquid chromatography system equipped with a G1311A quaternary pump and a G1312A fluorescence detector. LC separation was performed on a C_18_ Luna column (5 μm, 250 ´ 4.6 mm, Phenomenex, Torrance, CA, USA). The column compartment was kept at 25 °C. The mobile phase consisted of methanol:acetonitrile:water (15:18:67 *v*/*v*) at a flow rate of 1 mL/min. The fluorescence detector was set at 365 nm (excitation) and 455 nm (emission). The limit of quantification of AFB1 in the feeds was 1 μg/kg and the limit of detection was 0.3 μg/kg.

### 3.3. AFM1 Determination

Sample clean-up was carried out by the immunoaffinity technique, according to the AOAC official method [9]. Briefly, 50 mL of milk were warmed to 37 °C, centrifuged for 15 min at 4000 rpm and defatted. Then, 40 mL of the defatted fraction were passed through an immunoaffinity column (Aflaprep M, R-Biopharm Rhone, Glasgow, Scotland). The column was washed twice with 10 mL of phosphate-buffered saline followed by 10 mL of bi-distilled water, and eluted with 1.25 mL of methanol:acetonitrile (20:30 *v*/*v*), followed by 1.25 mL of bi-distilled water. The eluent was analyzed by LC/MS/MS. The method utilized an Agilent 1100 (Agilent Technologies, Böblingen, Germany) liquid chromatography system (equipped with binary pump, degasser, column compartment and autosampler) combined with an Applied Biosystems ABI 3200 QTrap (Applied Biosystems, Toronto, Canada) mass spectrometer. LC separation was performed using a C_18_ Hypersil Gold column (3 μm, 100 × 2.1 mm, Thermo Electron Corporation, Bellefonte, PA, USA). The mobile phase consisted of 0.2% formic acid (Sigma-Aldrich, St. Louis, MO, USA) and acetonitrile (J.T. Baker, Deventeer, The Netherlands). A linear gradient increased acetonitrile concentration from 20% to 80% between 0 and 1 min at a flow rate of 0.4 mL/min. The column compartment was kept at 25 °C and the injection volume was 15 μL. Turbo ion spray for ESI/MS/MS in positive ion mode was operated at a temperature of 500 °C. Multiple reaction monitoring (MRM) was applied. Precursor ion 329.1 *m*/*z* and product ions 273.2 and 229.2 *m*/*z* were assigned. The limit of quantification of AFM1 in milk was 0.01 μg/kg. 

### 3.4. Carry-Over Calculation

The carry-over of AFB1 to AFM1 in milk was calculated as the percentage of the AFB1 consumed that was excreted as AFM1 in the milk at the time when AFM1 output in milk had reached a steady state (Days 3 to 7). AFB1 concentrations in the feed were the sum of the added 80 μg AFB1 in the 500 g of corn meal and AFB1 residues in corn meal and the daily samples of TMR that were consumed. 

### 3.5. Statistical Analyses

The carry-over% was regressed on milk yield (kg) over time of collection (day) and the linear equation was calculated. The relationship between carry-over rate and the milk yield was assessed by Wilcoxon matched-pairs signed-ranks test.

## 4. Conclusions

In the current study, the mean carry-over at steady state was 2.5% for LL cows (yield < 35 kg/day) and 5.4% for ML cows (yield > 35 kg/day), thereby being within the ranges reported in previous studies [16,17,18,19,20,21,22]. When mean milk yield was paired with the median carry-over% in order to evaluate the correlation at steady state, an exponential regression displayed the best correlation, producing the equation:

carry-over% = 0.5154 e^0.0521*milk yield^, with *r*^2^ = 0.6224 (3)

In order to ensure milk production compliant with the regulatory MRL of 0.05 μg/kg AFM1 applied in Israel, the daily AFB1 exposure of the Israeli cow, characterized by an average daily intake of 25 kg DM feed and an average daily milk yield of 45 kg during early lactation [26,27], has to be below 1.4 μg/kg DM feed. It remains to be seen whether a regulatory change should be made to match our findings with the feed MRL according to the ALARA principle. 
